# Molecular characterization and impacts of a strain of *Grapevine leafroll-associated virus 2* causing asymptomatic infection in a wine grape cultivar

**DOI:** 10.1186/1743-422X-10-324

**Published:** 2013-10-30

**Authors:** Sudarsana Poojari, Olufemi J Alabi, Rayapati A Naidu

**Affiliations:** 1 Department of Plant Pathology, Washington State University, Irrigated Agriculture Research and Extension Center, Prosser, WA 99350, USA; 2 Current address: Department of Plant Pathology & Microbiology, Texas A&M AgriLife Research & Extension Center, 2401 East Highway 83, Weslaco, TX 78596, USA

## Abstract

**Background:**

Grapevine leafroll (GLD) is considered as the most economically important virus disease affecting wine grapes (*Vitis vinifera* L.) in many grapevine-growing regions. GLD produces distinct symptoms in red- and white-berried cultivars. In this study, we determined the complete genome sequence of an asymptomatic strain of *Grapevine leafroll-associated virus 2* (GLRaV-2) and studied its impacts on fruit yield and berry quality attributes in an own-rooted, red-berried wine grape cultivar.

**Findings:**

The complete genome of GLRaV-2 obtained from a red-berried wine grape cultivar Sangiovese, designated as GLRaV-2-SG, was determined to be 16,474 nucleotides in length. In pairwise comparisons, using complete genome sequences of GLRaV-2 strains available in GenBank, GLRaV-2-SG was more closely related to GLRaV-2-OR1 from Oregon, USA, and GLRaV-2-93/955 from South Africa, and distantly related to GLRaV-2-BD from Italy and GLRaV-2-RG from USA. Fruit yield estimates and berry quality analysis at the time of commercial harvest indicated that GLRaV-2-SG had little impact on fruit yield and total soluble solids, juice pH and total anthocyanins of berry skin.

**Conclusions:**

Because so little is known about the effects of asymptomatic virus infections in wine grapes, this study expanded our knowledge of the occurrence and impacts of GLRaV-2 causing asymptomatic infections. Our results indicated that an asymptomatic strain of GLRaV-2 may not cause significant effects to overall fruit yield and berry quality in own-rooted vines, but can affect its host in more subtle ways. Since disease symptoms are not apparent, relying on visual symptoms during disease surveys may result in the escape of asymptomatic strains of GLRaV-2. Thus, it is necessary to use appropriate diagnostic assays for reliable detection of viruses causing asymptomatic infections.

## Findings

Diseases caused by plant viruses are one of the major factors limiting sustainable production of wine grapes (*Vitis vinifera* L.) in many grapevine-growing regions [1,2]. Among them, grapevine leafroll (GLD) is considered as the most economically important disease [3]. GLD produces distinct symptoms in red- and white-berried cultivars [4] and is known to affect vine vigor, fruit yield and berry quality in both grafted and own-rooted plants [5-9]. A number of genetically distinct viruses, designated as grapevine leafroll-associated viruses (GLRaVs) and numbered serially as GLRaV-1, -2, -3, etc. in the order of their documentation, have been reported in grapevines affected with GLD [10]. All GLRaVs belong to the family *Closteroviridae*, with majority of them assigned to the genus *Ampelovirus*. In contrast, GLRaV-2 belongs to the genus *Closterovirus* and GLRaV-7 is assigned to the proposed genus *Velarivirus*. GLRaV-8 is no longer considered as a valid species in the family *Closteroviridae,* since the reported sequence was from the grapevine genome [10].

In addition to distinct taxonomic and genomic characteristics that set apart GLRaV-2 from other GLRaVs, the virus is known to occur as divergent molecular variants segregating into six distinct lineages [11]. GLRaV-2 variants have been implicated in a wide range of pathological properties, including leafroll, graft union-incompatibility on sensitive rootstocks, young vine decline and rootstock stem lesion disease [12-17]. A recent study indicated correlation between variants of GLRaV-2 belonging to distinct phylogenetic lineages and their pathological properties [16]. Most of these observations, however, were made with grafted vines using specific rootstocks. In contrast, the behavior of GLRaV-2 variants in own-rooted vines is not well studied.

In a recent study, molecular diversity of field isolates of GLRaV-2 collected from commercial vineyards in the Pacific Northwest region of the U.S. was reported [11]. Although most of these isolates were collected from own-rooted, red and white--berried wine grape cultivars showing GLD symptoms, some isolates were obtained from own-rooted Sangiovese vines showing no apparent visual symptoms. In this study, we determined the complete genome sequence of a GLRaV-2 isolate, designated as GLRaV-2-SG, causing asymptomatic infection in cv. Sangiovese, analyzed its genome characteristics with respect to other characterized GLRaV-2 strains and studied its impact on fruit yield and berry quality attributes.

Plant material was collected from Sangiovese vines planted in 1997 in a commercial vineyard. The name and location of this block was withheld to maintain grower confidentiality. Scrapings of cambial tissues from hardwood cuttings, obtained from a single grapevine tested positive only for GLRaV-2 ([11] Unpublished results) were used for isolating genomic-length, double-stranded (ds) RNA as described by Valverde et al. [18]. The dsRNA-enriched preparation was subjected to DNase I (Invitrogen, Carlsbad, CA, USA) treatment and subsequently used as a template for amplification of different portions of the virus genome.

Consensus primers were designed based on alignment of nucleotide sequence of the complete genome of GLRaV-2 strains available in GenBank (Additional file 1: Table S1). One step-single tube RT-PCR assays were carried out to amplify overlapping segments of the virus genome using the following conditions: cDNA synthesis at 50°C for 45 min followed by initial denaturation at 95°C for 5 min, 40 consecutive cycles of 95°C for 30 s, 57°C for 60 s and 72°C for 90 s per cycle, and a final extension step for 10 min at 72°C. The amplicons were cloned into pCR2.1 vector (Life Technologies, Carlsbad, CA, USA) and three independent clones per amplicon were sequenced in both orientations. Wherever necessary, additional clones were sequenced to resolve nucleotide sequence ambiguities. The 5′ and 3′ terminal sequence of GLRaV-2 was determined using the FirstChoice® RLM-RACE Kit (Ambion, Austin, TX, USA) following the manufacturer's instructions.

GLRaV-2-specific primers (SG5p: 5′-GAGCGACTGAGCAGGGAAGGGTGG-3′ and SG3p: 5′-GAGCGACTGAGCAGGGAAGGGTGG-3′) were used in combination with primers provided in the RACE kit to amplify approximately 150 bp and 250 bp fragments corresponding to the 5′ and 3′ termini, respectively. Amplicons were purified using the QIAquick PCR purification kit (Qiagen Inc., Valencia, CA, USA) following the manufacturer’s instructions, cloned and 15 recombinant clones per amplicon sequenced in both orientations. Nucleotide sequence annotation, ORF prediction and sequence assembly were carried out using Vector NTI Advance 11 program (Life Technologies, Carlsbad, CA, USA). Multiple alignments of nucleotide and amino acid sequences and construction of Neighbor-Joining (NJ) phylogenetic trees were performed using ClustalW with default settings from MEGA5 software [19].

To study impacts of GLRaV-2-SG, five pairs of own-rooted Sangiovese vines, with each pair consisting of GLRaV-2-SG-infected and non-infected grapevines located adjacent to each other in a given row, were selected to minimize error in experimental results due to variations in soil and other growing conditions. At the time of commercial harvest in October 2012, berry clusters from individual grapevines were counted and their combined weight measured. Juice extracted from 100 berries collected randomly from individual GLRaV-2-SG-infected and non-infected vines was used separately to measure total soluble solids (TSS), pH, titratable acidity (TA) and total anthocyanins as described previously [20]. Statistical significance of the data between GLRaV-2-SG-infected and non-infected vines was analyzed by One-way ANOVA. Data are presented as mean ± SE (standard error). The minimum accepted *P* value for significance was 0.05.

**Figure 1:**
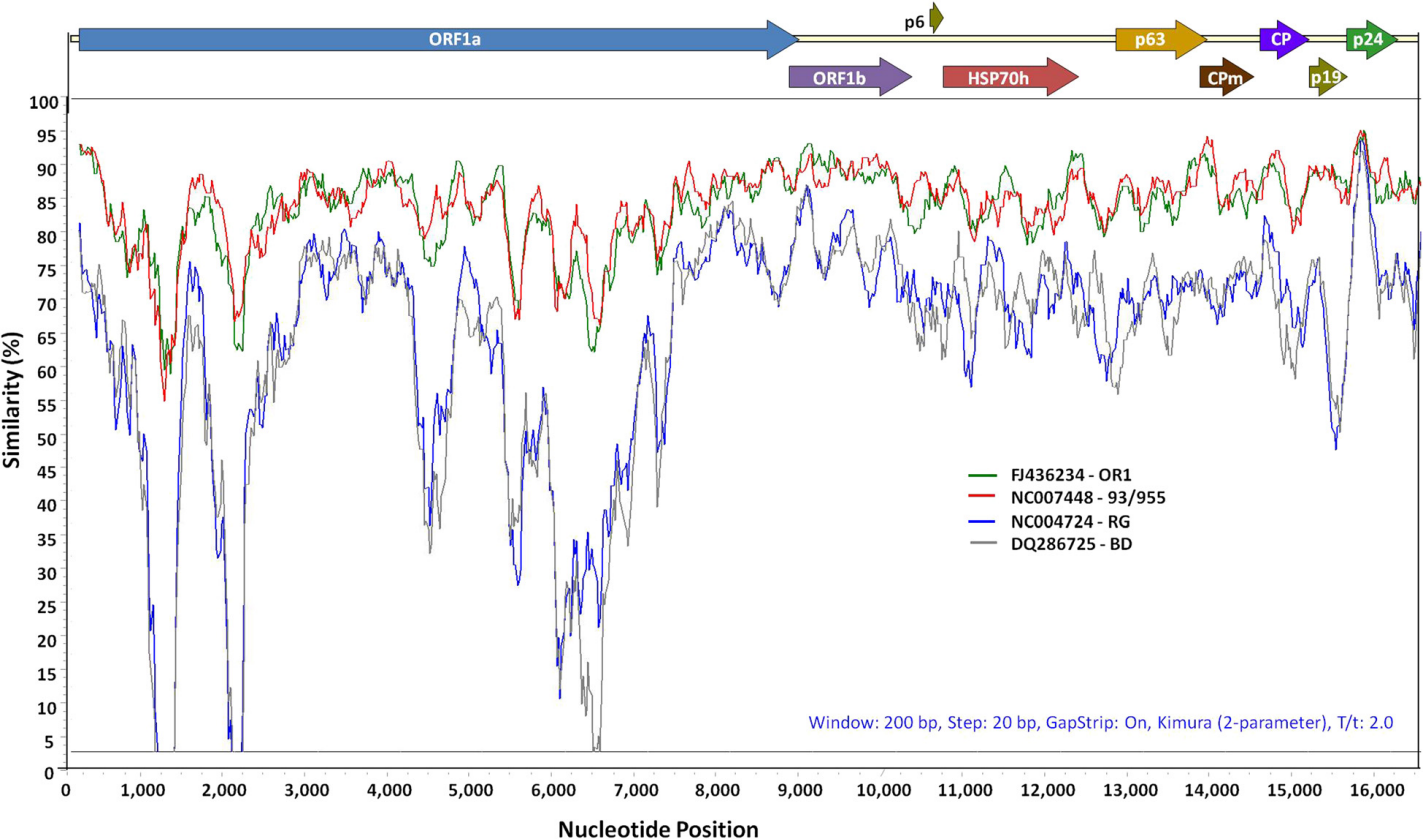
**Sliding-window Simplot graph showing genome-wide comparison of *Grapevine leafroll-associated virus 2, *strain SG (GLRaV-2-SG) with other strains of GLRaV-2 (OR1, 93/955, BD and RG; GenBank: FJ436234; NC007448; DQ286725; NC004724).** The Simplot was generated using multiple sequence alignment of all strains with a window size of 200 nt and a step size of 20 nts. Diagrammatic representation of the GLRaV-2 genome with location of 9 open reading frames (ORFs) from 5′ to 3′ are shown above the graph. See Martelli et al. [10] for details of genome organization of the virus.

Using the dsRNA-enriched preparation from a single asymptomatic Sangiovese vine as a template (Additional file 1: Figure S1), we amplified overlapping cDNA fragments spanning the entire genome of the virus with primers listed in Additional file 1: Table S1. By cloning and sequencing of amplicons, we assembled the 16,474 nucleotide (nt) genome of GLRaV-2-SG isolate (GenBank: KF220376). Our analysis indicated that the organization of GLRaV-2-SG genome is similar to other characterized strains of GLRaV-2 and encodes nine putative ORFs with 106 nt long 5′ non-translated region (NTR) and 206 nt long 3′NTR (Figure 1 and Table 1). A pairwise comparison of the complete genome sequence of GLRaV-2-SG with other GLRaV-2 sequences available in GenBank indicated that GLRaV-2-SG shares 85% sequence identity with GLRaV-2-93/955 (NC007448) from South Africa and GLRaV-2-OR1 (FJ436234) from Oregon, USA, and 72% identity with GLRaV-2-BD (DQ286725) from Italy and GLRaV-2-RG (NC004724) from USA. A pairwise comparison of individual ORFs encoded by GLRaV-2-SG with corresponding sequences from other isolates are shown in Table 1. The data indicated greater than 86% amino acid (aa) sequence identities among all ORFs, except p19 that showed 83 and 84% aa identity with corresponding sequence of isolates OR1 and 93/955 and 71% identity with BD and RG isolates. The regions with the lowest nt sequence identity were identified within ORF1a between nt 950–1193, nt 1575–2196 and nt 5769–6828. GLRaV-2-SG shared 78-89% aa sequence identity with other GLRaV-2 isolates (OR1, 93/955, BD and RG) (Figure 1) in these three regions resulting in significant aa sequence differences (data not shown). Since the aa identity values of the ORF1b, p6, heat-shock protein 70 homolog (HSP70h), p63, coat protein (CP), minor coat protein (CPm) and p24, with the exception of p19, fall within the 10-15% identity range, GLRaV-2-SG may be considered as a distinct strain of GLRaV-2 based on the criteria established for sequence identity threshold values suggested for demarcation of species and their strains of GLRaVs [10].

**Table 1:** **Percent (%) nucleotide (nt) and amino acid (aa) identities between non-translated regions, protein-coding genes and complete genome of** SG strain ***Grapevine leafroll-associated virus 2*** **(GLRaV-2-SG; KF220376) and other strains of GLRaV-2 (OR1, 93/955, BD and RG; GenBank: FJ436234; NC007448; DQ286725; NC004724)**

**GLRaV-2-SG**	**Length (bp)**	**% Identity**							
		**OR1**		**93/955**		**BD**		**RG**	
		**nt**	**aa**	**nt**	**aa**	**nt**	**aa**	**nt**	**aa**
5′ NTR	106	94	--	96	--	86	--	83	--
ORF1a	8808	84	86	84	87	69	71	70	72
ORF1b	1263	88	96	89	98	79	94	79	94
p6	171	87	95	87	91	75	77	78	78
HSP70h	1662	86	91	86	90	76	86	76	84
p63	1656	86	90	88	92	75	80	74	80
CPm	672	86	94	87	96	78	90	77	87
CP	597	87	92	88	92	75	84	76	87
p19	483	90	83	90	84	76	71	75	71
p24	618	87	93	88	92	77	81	78	81
3′ NTR	205	88	--	85	--	83	--	83	--
Complete genome	16474	85	--	85	--	72	--	72	--

Fruit yield measurements at the time of commercial harvest indicated no significant differences in the number of clusters and cumulative weight of fruits between GLRaV-2-SG-positive and negative Sangiovese vines (Table 2). Analysis of fruit quality parameters indicated that TSS (measured as °Brix) and TA was reduced by 3.2% and 3.8%, respectively, in berries from vines infected with GLRaV-2-SG, compared to berries from virus-negative vines. Likewise, no significant differences were observed in berry juice pH between virus-positive and virus-negative vines. Total extractable anthocyanins of berries were less by 17.7% in virus-positive vines compared to adjacent virus-negative vines. However, the difference was considered statistically insignificant due to high standard deviation that is more than three quarters of the value of the difference between virus-positive and negative samples. Additional studies involving more sampling size are in progress to resolve this discrepancy. Overall, these results suggest that GLRaV-2-SG showed no significant negative impacts on fruit yield and berry quality in infected vines.

**Table 2:** **Impact of SG strain *Grapevine leafroll-associated virus 2 *** **(GLRaV-2-SG) on fruit yield and berry quality attributes**

**Sample**	**Yield**		**Berry biochemical analysis**			
	**Avg. number of berry clusters**	**Avg. weight of berry clusters**	**Total soluble solids** **(˚Brix)**	**TA ** **(g/L)**	**pH**	**Total anthocyanins**
						**(mg/g berry wt.)**
GLRaV-2-SG-positive	14.66 ± 0.55	4035 ± 281	24.540 ± 0.10	7.87 ± 0.04	3.43 ± 0.01	0.84 ± 0.03
GLRaV-2-SG-negative	12.83 ± 0.87	3950 ± 528	25.34 ± 0.10	7.57 ± 0.27	3.48 ± 0.01	1.02 ± 0.11

In conclusion, this study described the molecular characterization of a naturally occurring GLRaV-2 strain causing asymptomatic infection in an own-rooted wine grape cultivar Sangiovese. GLRaV-2-SG infection with no visually apparent symptoms caused insignificant impacts on yield and fruit quality. A recent study found asymptomatic infections of GLRaV-7 in wine grape cultivars, such as Pinot Noir and Cabernet Franc [21]. Although the mechanism(s) behind asymptomatic infection of GLRaVs has yet to be elucidated, our results, together with observations reported by Al Rwahnih et al. [21] for GLRaV-7, support the possibility of the occurrence of GLRaVs causing asymptomatic infections in wine grape cultivars. Further research is in progress to study if the asymptomatic phenotype of GLRaV-2-SG is an intrinsic property of the virus or dependent on specific virus-cultivar interactions. Regardless, this study provides further evidence that visual symptoms alone are not a reliable criterion for the field diagnosis of GLD and it is more likely that GLRaVs causing asymptomatic infections can be disseminated via vegetative propagation. Thus, rigorous testing of grapevines is vital to prevent escape of asymptomatic strains through the supply of planting materials to nurseries and grape growers.

## Abbreviations

RdRp: RNA-dependent RNA polymerase; HSP70h: Heat-shock protein 70 homolog; CP: Coat protein; CPm: Minor coat protein.

## Competing interests

The authors have declared that no competing interests exist.

## Authors’ contributions

Conceived and designed the experiments: RAN, SP, OJA. Performed the experiments: SP, OJA. Analyzed the data: SP, OJA, RAN. Contributed reagents/materials/analysis tools: RAN. Wrote the paper: SP, OJA, RAN. All authors read and approved the final manuscript.

## Supplementary Material

Additional file 1: Figure S1 Electrophoretic pattern of dsRNA-enriched preparation of *Grapevine leafroll-associated virus 2*, strain SG (GLRaV-2-SG) obtained from cv. Sangiovese (lane 2). dsRNA-enriched preparation from *Nicotiana benthamiana* infected with *Potato virus* Y (PVY) and *Cucumber mosaic virus* (CMV) (lane 1) were included as markers. Expected size of dsRNAs is shown by arrows. M represents 1Kb Plus DNA ladder (Life Technologies, Carlsbad, CA, USA). **Table S1.** Nucleotide sequences of forward (F) and reverse (R) primers used for amplification of the complete genome of GLRaV-2-SG. Position refers to the location of primer sequences on the GLRaV-2-SG genome. Click here for file
