# Supplementary figures and images for: Molecular characterization and impacts of a strain of Grapevine leafroll-associated virus 2 causing asymptomatic infection in a wine grape cultivar
Source: Virol J. 2013 Oct 30;10:324. doi: 10.1186/1743-422X-10-324 (PMC3828392; doi:10.1186/1743-422X-10-324)

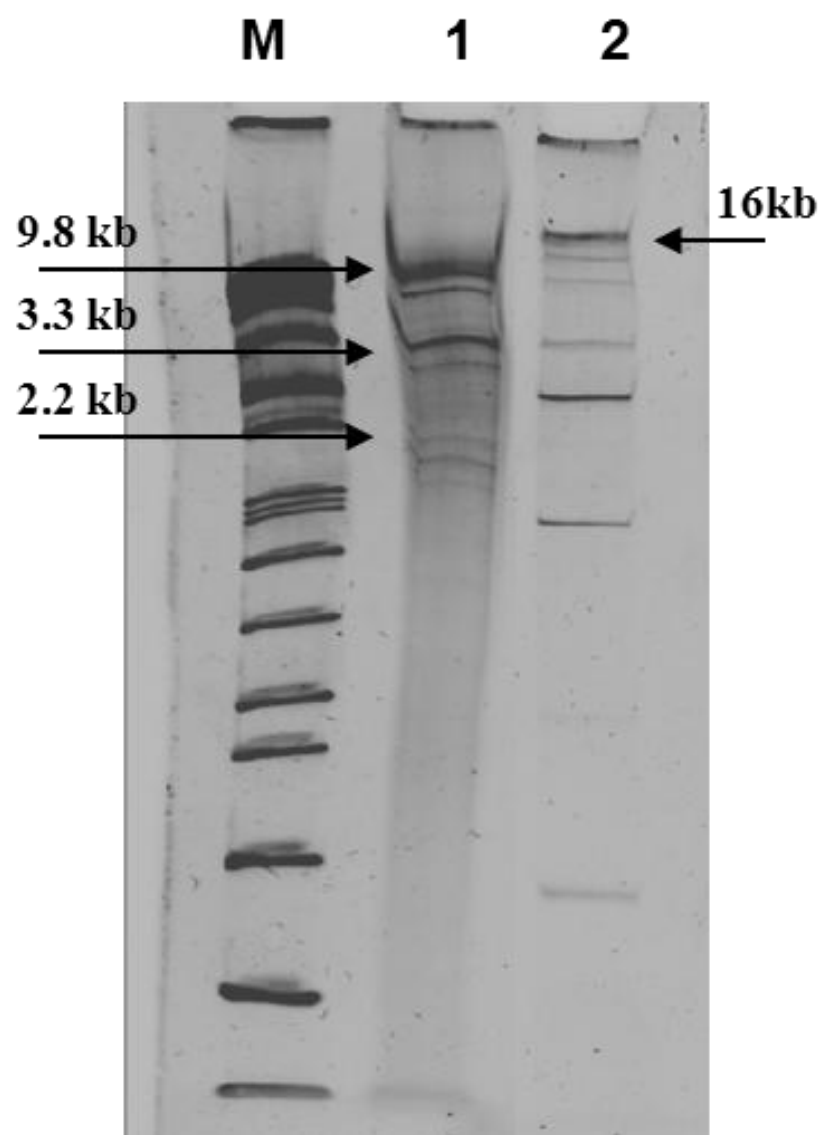

Supplement: Additional file 1: Figure S1 — Electrophoretic pattern of dsRNA-enriched preparation of Grapevine leafroll-associated virus 2, strain SG (GLRaV-2-SG) obtained from cv. Sangiovese (lane 2). dsRNA-enriched preparation from Nicotiana benthamiana infected with Potato virus Y (PVY) and Cucumber mosaic virus (CMV) (lane 1) were included as markers. Expected size of dsRNAs is shown by arrows. M represents 1Kb Plus DNA ladder (Life Technologies, Carlsbad, CA, USA). Table S1. Nucleotide sequences of forward (F) and reverse (R) primers used for amplification of the complete genome of GLRaV-2-SG. Position refers to the location of primer sequences on the GLRaV-2-SG genome. [file 1743-422X-10-324-S1.pdf]
